# Demographic and Geographic Differences in Exposure to Secondhand Smoke in Missouri Workplaces, 2007-2008

**Published:** 2011-10-15

**Authors:** Jenine K. Harris, Caroline Geremakis, Sarah Moreland-Russell, Bobbi J. Carothers, Sarah C. Shelton, Barbara Kariuki, Matthew Kuhlenbeck

**Affiliations:** George Warren Brown School of Social Work, Washington University in St. Louis. Dr Harris is also affiliated with the Center for Tobacco Policy Research, Washington University in St. Louis, Saint Louis, Missouri; School of Public Health, Saint Louis University, Saint Louis, Missouri; Center for Tobacco Policy Research, Washington University in St. Louis, Saint Louis, Missouri; Center for Tobacco Policy Research, Washington University in St. Louis, Saint Louis, Missouri; Center for Tobacco Policy Research, Washington University in St. Louis, Saint Louis, Missouri; School of Medicine, Saint Louis University, Saint Louis, Missouri; Matthew Kuhlenbeck, Missouri Foundation for Health, Saint Louis, Missouri

## Abstract

**Introduction:**

African Americans, Hispanics, service and blue-collar workers, and residents of rural areas are among those facing higher rates of workplace secondhand smoke exposure in states without smokefree workplace laws. Consequently, these groups also experience more negative health effects resulting from secondhand smoke exposure. The objective of this study was to examine disparities in workplace secondhand smoke exposure in a state without a comprehensive statewide smokefree workplace law and to use this information in considering a statewide law.

**Methods:**

We developed a logistic multilevel model by using data from a 2007-2008 county-level study to account for individual and county-level differences in workplace secondhand smoke exposure. We included sex, age, race, annual income, education level, smoking status, and rural or urban residence as predictors of workplace secondhand smoke exposure.

**Results:**

Factors significantly associated with increased exposure to workplace secondhand smoke were male sex, lower education levels, lower income, living in a small rural or isolated area, and current smoking. For example, although the overall rate of workplace exposure in Missouri is 11.5%, our model predicts that among young white men with low incomes and limited education living in small rural areas, 40% of nonsmokers and 56% of smokers may be exposed to secondhand smoke at work.

**Conclusion:**

Significant disparities exist in workplace secondhand smoke exposure across Missouri. A statewide smokefree workplace law would protect all citizens from workplace secondhand smoke exposure.

## Introduction

Progress has been achieved in the United States during the past 20 years in establishing smokefree environments in homes, workplaces, and public places such as restaurants and bars. Approximately 70% of workers in the United States are now protected by a smokefree workplace policy (1), and the proportion of nonsmokers nationally with detectable cotinine levels (a biomarker for secondhand smoke exposure) has been halved from 88% to 43%, meeting the *Healthy People 2010* objective in this area (2,3).

Despite this success, significant disparities in secondhand smoke exposure persist. African Americans are more heavily exposed to secondhand smoke than whites and Mexican Americans (4). People with lower incomes are more heavily exposed than those with higher incomes (4-7). Certain categories of workers, including blue collar, service, and hospitality workers, are substantially less likely to be protected by a smokefree workplace law and are substantially more likely to be exposed to secondhand smoke on the job than white collar and professional workers (8-11). Nonsmoking men, younger adults, and those living in a county without any smokefree workplace restrictions are substantially more likely to be exposed to secondhand smoke (12,13) than nonsmoking women, older adults, and people living in counties with some workplace smoking restrictions. In addition to being exposed more often to secondhand smoke, these groups are also disproportionately burdened by the poor health outcomes associated with tobacco use and secondhand smoke exposure (14).

Exposure to secondhand smoke can be decreased by implementing comprehensive state and local smokefree workplace laws (2,12,15-17). Comprehensive statewide laws protect the entire state, including people who have traditionally faced disproportionate exposure to secondhand smoke and health disparities related to tobacco use (18). These laws can be especially beneficial for disadvantaged groups, leading to a reduction in disparities (19,20).

Statewide laws have also demonstrated effectiveness in reducing the negative health consequences of secondhand smoke exposure (21). For example, following enactment of New York's statewide smokefree workplace law in 2003, by 2004 the number of myocardial infarctions in New York decreased by 7.9%, and the number of strokes decreased by 11% (21,22).

Missouri does not have a comprehensive statewide smokefree workplace law and ranks 50th (of the 50 states and the District of Columbia) in the percentage of indoor employees exposed to secondhand smoke — 12% compared with 7.3% nationwide (23). The objectives of this study were to 1) identify the characteristics of Missourians most affected by secondhand smoke exposure in the workplace, and 2) describe geographic variation in exposure to secondhand smoke statewide. A secondary objective was to examine the support for a statewide smokefree law among Missourians.

## Methods

### Data collection

We conducted a descriptive cross-sectional study by using survey data collected in the 2007 Missouri County-Level Study (CLS). The Missouri Department of Health and Senior Services and the Missouri Foundation for Health conducted the CLS to determine county-level prevalence of behavioral risk factors, chronic diseases, and preventive practices among adults (www.health.mo.gov/data/cls/). Survey administration followed Centers for Disease Control and Prevention (CDC) Behavioral Risk Factor Surveillance System (BRFSS) methods; the survey consisted of items from the BRFSS and CDC Adult Tobacco Survey. Data were collected from February 2007 through April 2008 via telephone interviews of adults aged 18 years or older. The response rate was 60.3% based on response rate formula 2 (RR2) from the American Association of Public Opinion Research (24).

There were 49,513 completed interviews; no information was collected on nonrespondents. Of the 49,513 participants, 30,398 indicated that they were currently employed for wages or self-employed; 23,923 indicated that they worked indoors most of the time, and 23,820 (99.6%) of indoor workers responded to the question assessing secondhand smoke in the workplace. We conducted analyses on these 23,820 participants. Data were weighted to be representative of the Missouri adult noninstitutionalized population based on distributions of age, sex, race, and county of residence from the 2000 census.

### Measures

We selected the following individual-level characteristics to study: sex, age, race, annual income, education level, and smoking status. A review of studies on the reliability and validity of BRFSS measures found age, sex, race/ethnicity, education, income, and smoking status to have high reliability and moderate to high validity (25). Although occupation has been identified as a factor in secondhand smoke exposure, it was not assessed in the CLS. Age was measured in years. Race was identified by 4 categories: non-Hispanic white, non-Hispanic black, Hispanic, or other. Annual income was measured in 6 increments, starting with less than $15,000 as the lowest category and $75,000 or more as the highest category. Income was treated as continuous in the models. Education was categorized into less than high school graduate, high school graduate or General Educational Development certificate, some college, or college graduate. Smoking status was collapsed from current, former, and never smoker status into current smoker and nonsmoker. The county-level predictor was rurality, and was determined by the Rural-Urban Continuum Codes (RUCC) (www.ers.usda.gov/briefing/rurality/ruralurbcon/), which we classified into 4 categories (urban, large rural, small rural, or isolated).

Dependent variables were workplace secondhand smoke exposure and support for a smokefree workplace law. The workplace secondhand smoke exposure variable was measured by determining how many participants answered yes to both of the questions "While working at your job, are you indoors most of the time?" and "As far as you know, in the past 7 days, has anyone smoked in your work area?" Support for a smokefree workplace law was measured by response to the question "Some cities and towns are considering laws that would make workplaces smokefree by prohibiting smoking in all indoor workplaces, including restaurants, bars and casinos. Would you support such a law in your community?" Even though these questions are specific to support of smokefree workplaces laws at the local level, support for local laws can increase support and demand for a statewide law by increasing awareness, demonstrating the ease of implementation, and changing social norms (23,26). Perceptions of the health effects of secondhand smoke was measured by response to the item, "Do you think that breathing smoke from other people's cigarettes is: 1-Very harmful to one's health; 2-Somewhat harmful to one's health; 3-Not very harmful to one's health; and 4-Not harmful at all to one's health." The variable was collapsed into a binary variable where yes represented responses 1 and 2 and no represented responses 3 and 4.

People with missing data for any of the independent variables were not included in the final model. We used HLM 6.08 for Windows (Scientific Software International, Inc, Lincolnwood, Illinois) for multilevel modeling and SPSS 17.0.1 (SPSS, Inc, Chicago, Illinois) for all other analyses.

### Analyses

We employed bivariate statistics to describe the sample and examine differences in workplace secondhand smoke exposure related to geographic and demographic characteristics. To determine whether a multilevel modeling strategy was appropriate, we used 2 strategies to test the heterogeneity of proportions, that is, does workplace secondhand smoke exposure vary significantly by county? First, we calculated an intraclass correlation coefficient (ICC [ρ]) specifically developed for binary outcomes (27) between workplace secondhand smoke exposure and county of residence. Second, we conducted a χ^2^ test of county and workplace secondhand smoke exposure (28). In addition to examining the significance of the χ^2^ statistic, we examined the standardized residuals to determine any significant differences. Standardized residuals with a magnitude greater than 2 are major contributors to significant χ^2^ results; they indicate that the observed frequency was much higher than expected, and standardized residuals less than −2 indicate that the observed frequency was much lower than expected.

Although we found a low ICC value (ρ_workplace_ = 0.014), the χ^2^ analyses found significant differences across Missouri counties for exposure to secondhand smoke in the workplace (χ^2^(114) = 334.4; P < .001). Because of this heterogeneity of proportions, we used multilevel modeling to examine secondhand smoke exposure in Missouri, accounting for individual and county-level differences. Specifically, we developed multilevel logistic regression random intercepts models consisting of the following: 1) null model, 2) individual-level characteristics (model 1), and 3) model 1 plus county-level characteristics (model 2). We assessed measures of model significance (χ2) and model fit (Akaike Information Criterion [AIC], deviance) and compared models by using the likelihood ratio (LR) test. AIC and deviance are used with nested models to quantify the lack of fit in 1 model relative to the other; lower AIC values represent better fitting models with less lack of fit. LR tests are used with nested logistic models to determine which model is a better fit. The LR statistic is the difference between the measures of deviance (lack of fit) of 2 models, and it follows a χ^2^ distribution with degrees of freedom equal to the difference between the parameters in the 2 models (29). A significant LR test indicates that the larger model is a better fit. As a demonstration, we calculated the probability of secondhand smoke exposure in specific groups of Missourians by substituting values representing the groups into our final model.

Finally, to determine the feasibility of adopting and implementing a statewide smokefree workplace law in Missouri, we conducted bivariate analyses to examine support for a workplace law across demographic and geographic groups. Where there were significant differences, we examined standardized residuals to determine which groups were significantly different in terms of their support for a smokefree law.

## Results

### Workplace secondhand smoke exposure

**Figure 1.**
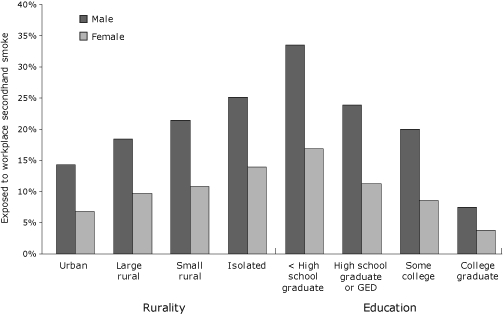
Percentage of men and women employed indoors and exposed to workplace secondhand smoke, Missouri County-Level Study, 2007-2008. Abbreviation: GED, General Educational Development certificate. Rurality was determined by using the Rural-Urban Continuum codes (www.ers.usda.gov/briefing/rurality/ruralurbcon/).

Of the 23,820 indoor workers who responded to the question assessing secondhand smoke in the workplace, 2,740 respondents (11.5%) reported being exposed to secondhand smoke at work in the last week. Extrapolated to more than 2 million Missourians employed in indoor settings, this finding indicates that approximately 236,000 Missourians are exposed to secondhand smoke in their workplaces. Demographic characteristics of those exposed and not exposed to secondhand smoke at work are shown in Table 1, and a few of the notable differences are demonstrated in Figure 1. Among those exposed to secondhand smoke in the workplace, 60.9% were nonsmokers, and 88.9% believed secondhand smoke exposure was harmful to health; however, 62.0% declared that they were not in favor of a local smokefree workplace law. Close to half (41.2%) of those not in favor were current smokers (data not shown). There was a significant association between smoking status and support for a local workplace law among those who were exposed (χ^2^(1) = 419.7; *P* < .001); 13.7% of current smokers and 53.7% of nonsmokers supported a workplace law. Support for a local workplace law was high (60.3%) among people who had not been exposed to secondhand smoke at work.

**Table:** 

**Characteristic**	**Men**	**Women**
**Rurality**		
Urban	14%	7%
Large rural	19%	10%
Small rural	22%	11%
Isolated	25%	14%
**Education**		
<High school	34%	17%
High school or GED	24%	11%
Some college	20%	9%
College graduate	8%	4%

**Figure 2.**
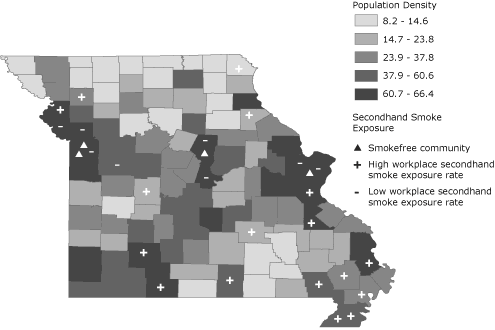
Population density per square mile by county (2000 US Census), exposure to workplace secondhand smoke (2007-2008 Missouri County-Level Study [CLS]), and location of communities that enacted comprehensive smokefree ordinances just before collection of CLS data. Plus and minus signs indicate counties with much higher or lower than expected workplace secondhand smoke exposure. See the Methods section for more details of this analysis.

Exposure to secondhand smoke varied in counties across the state. A few of the counties with lower than expected secondhand smoke exposure (standardized residuals less than −2) have communities that enacted comprehensive smokefree workplace ordinances shortly before the CLS data were collected (Figure 2). All of the counties with lower than expected secondhand smoke exposure had high population densities and were clustered around the 3 largest metropolitan areas, Kansas City, Columbia, and Saint Louis. Counties with higher than expected secondhand smoke exposure rates were scattered throughout the state, but were particularly concentrated in the southeast boot-heel region, which is mostly rural.

**Figure 3.**
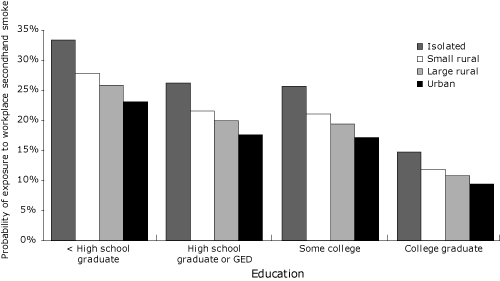
The probability of secondhand smoke exposure at work among white male nonsmokers in Missouri, by education and rurality (average age, 41.7 y; median annual income, $35,000-$49,999). Probabilities are based on the model presented in Table 2. Area of residence (rurality) was determined by using the Rural-Urban Continuum codes (www.ers.usda.gov/briefing/rurality/ruralurbcon/). Abbreviation: GED, General Educational Development certificate.

All 3 models demonstrated statistical significance. A comparison of fit indices (deviance and AIC) indicated that the model including individual and county predictors was the best fit. LR tests comparing model 1 to the null model and comparing model 2 to model 1 yielded significant results. Given the measures of fit and significant LR test (χ^2^(3) = 18.0; *P* = .001), model 2 was adopted as the final model (Table 2). Age, sex, income, education, smoking status, and rurality were all predictors of the probability of secondhand smoke exposure. Race, however, was not. Living in a small rural or isolated area significantly increased the likelihood of exposure to secondhand smoke at work compared with living in an urban setting. However, living in a large rural area was not significantly different than living in an urban setting. Increased age and increased income were associated with a reduced likelihood of secondhand smoke exposure in the workplace. Male sex, lower levels of education, and current smoking were significantly associated with increased secondhand smoke exposure. The more rural the county a person lived in and the less education they had, the more likely they were to be exposed to secondhand smoke at work (Figure 3).

**Table:** 

**Education Level**	**Isolated**	**Small rural**	**Large rural**	**Urban**
<High school graduate	33%	28%	26%	23%
High school graduate or GED	26%	21%	20%	18%
Some college	26%	21%	19%	17%
College graduate	15%	12%	11%	9%

Overall exposure to secondhand smoke at work across Missouri is 11.5%. However, among young white men with low incomes and limited education living in small rural areas, 40% of nonsmokers and 56% of smokers may be exposed to secondhand smoke at work. On the basis of our model, the highest exposure category is smokers who were young black men making less than $15,000 a year with less than a high school education and living in isolated areas. This group has a 66% chance of being exposed to secondhand smoke in the workplace. This same group with nonsmoking status would have a 50% chance of exposure based on our model.

### Support for a smokefree workplace law

Among employed Missourians, there were significant differences in the proportion of respondents supporting a smokefree workplace law by sex (χ^2^(1) = 113.3; *P* < .001), education level (χ^2^(3) = 528.2; *P* < .001), income category (χ^2^(5) = 78.3; *P* < .001), race (χ^2^(3) = 41.5; *P* < .001), exposure to secondhand smoke at work (χ^2^(1) = 468.9; *P* < .001), and smoking status (χ^2^(1) = 3,426.2; *P* < .001). There were no significant differences (*P* > .05) in support for a local smokefree law by age or rurality. Overall, 58% of Missourians support a local smokefree workplace law, regardless of exposure and smoking status.

Residual analyses indicated that, although our multilevel model showed that the following groups were more likely to be exposed to secondhand smoke, they were also significantly less likely than expected (standardized residuals less than −2) to support a local smokefree workplace law: men, smokers, people making less than $75,000 per year, and people with less than a college education. Respondents exposed to secondhand smoke in the workplace were also significantly less likely than expected to support local smokefree workplace laws. Conversely, many of those groups who were already protected (ie, women, college graduates, people with annual incomes of more than $75,000, people not exposed to secondhand smoke at work, and nonsmokers) were more likely than expected to indicate support for a local smokefree workplace laws. Race was not a significant predictor of secondhand smoke exposure in the multilevel model; however, there were a few notable differences in the bivariate analyses. Specifically, Hispanic and other race Missourians were more likely than expected to support local smokefree workplace laws.

## Discussion

The objectives of this study were to identify the demographic and geographic characteristics of Missourians exposed to secondhand smoke in the workplace. In addition, we sought to determine support for smokefree workplace laws among Missouri residents. Consistent with other studies of secondhand smoke exposure (4-13), we found in Missouri that exposure was significantly more likely for men, younger adults, those with less education, lower income people, current smokers, and residents of rural or isolated geographic areas. Although significant differences in exposure were shown by race in the bivariate analyses and have been identified in previous studies, race was not a significant predictor in the multivariate model. This may be due to the geographic distribution of race in Missouri; 94.6% of non-Hispanic black Missourians live in urban areas, where we found significantly lower levels of exposure to secondhand smoke. Finally, most Missourians support smokefree workplace laws; significantly higher levels of local law support were seen among nonsmokers, women, college graduates, those with high incomes, and people not exposed to secondhand smoke at work.

### Limitations

CDC considers people of low socioeconomic status and African American, Hispanic/Latino, Asian American/Pacific Islander/Native Hawaiian, American Indian/Alaska Native, and lesbian/gay/bisexual/transgender populations as facing tobacco-related disparities. The largest limitation of this study is the exclusion of the last 3 of these populations. In addition, the sample of Missourians analyzed here did not include residents with wireless telephone numbers only (30) or no telephone service at all (31). Recent studies have shown some demographic and health-related differences between people who use only a cellular telephone and those who use a land-based telephone line. People not reachable during the calling hours may represent important segments of the workforce, such as the service industry who are exposed to secondhand smoke. Because the location of the participant's employment was not collected, we were also unable to account for the existence of local smokefree laws in our analysis. However, given the very small number of cities (n = 4) that had already adopted comprehensive smokefree workplace laws at the time of data collection, we do not think that this omission affected our results. Although the data were weighted to be representative of the general population, the distribution of income was skewed toward higher income levels, limiting generalizability. However, because exposure to secondhand smoke is more common in lower income levels, we believe this skew may have precipitated an underestimation of the number of Missourians exposed to secondhand smoke at work.

### Recommendations

Although comprehensive smokefree workplaces policies adopted in some Missouri communities appear to have decreased secondhand smoke exposure on a local level, many areas in Missouri have been reluctant to adopt comprehensive smokefree workplace laws, allowing disparities to persist throughout the state. For example, St. Louis County implemented a smokefree workplace law on January 2, 2011 (32), but the law is not comprehensive. Exemptions leave those who work in small bars, casinos, hotels and motels, and several other venues at risk for workplace secondhand smoke exposure. Statewide comprehensive law is the only method that will ensure that those most at risk in Missouri are protected (33-35) and, on the basis of prior research, will reduce the overall rate of workplace secondhand smoke exposure by more than half (19). This reduction would provide an additional 133,000 Missourians with smokefree workplaces. Given the evidence related to exposure rates and disparities and the support for a smokefree law among Missourians, it may be a good time for policy advocates to push for a statewide comprehensive smokefree workplace law.

## Figures and Tables

**Table 1:** Demographic Characteristics and Exposure to Secondhand Smoke in the Workplace for Employed Participants Working Indoors, Missouri County-Level Study, 2007-2008

**Characteristic**	Exposed^a^	Unexposed^a^	*P* Value
**Age (n = 23,636), mean (SD), y**	39.3 (12.8)	42.0 (12.4)	<.001
**Sex (n = 23,820)**			
Male	1,737 (63.4)	9,274 (44.0)	<.001
Female	1,003 (36.6)	11,806 (56.0)	
**Race (n = 23,676)**			
White, non-Hispanic	2,307 (84.4)	17,982 (85.9)	.008
Black, non-Hispanic	321 (11.7)	2,213 (10.6)	
Hispanic	63 (2.3)	345 (1.6)	
Other	42 (1.5)	403 (1.9)	
**Education (n = 23,795)**			
<High school graduate	248 (9.1)	756 (3.6)	<.001
High school graduate or General Educational Development certificate	1,065 (38.9)	5,259 (25.0)	
Some college	853 (31.1)	5,516 (26.2)	
College graduate	574 (20.9)	9,524 (45.2)	
**Rurality^b^ (n = 23,674)**			
Urban	1,870 (68.6)	16,303 (77.8)	<.001
Large rural	195 (7.2)	1,218 (5.8)	
Small rural	465 (17.1)	2,550 (12.2)	
Isolated	196 (7.2)	877 (4.2)	
**Annual income (n = 21,854)**			
<$15,000	170 (6.9)	482 (2.5)	<.001
$15,000-$24,999	380 (15.3)	1,712 (8.8)	
$25,000-$34,999	364 (14.7)	1,898 (9.8)	
$35,000-$49,999	460 (18.6)	3,635 (18.8)	
$50,000-$74,999	510 (20.6)	4,568 (23.6)	
≥$75,000	592 (23.9)	7,083 (36.6)	
**Smoking status (n = 23,764)**			
Nonsmoker	1,663 (60.9)	16,794 (79.8)	<.001
Current smoker	1,066 (39.1)	4,241 (20.2)	
**Think secondhand smoke is harmful to health (n = 22,936)**			
Yes	2,321 (88.9)	18,775 (92.4)	<.001
No	290 (11.1)	1,550 (7.6)	
**Would support a smoke-free workplace law (n = 22,598)**			
Yes	990 (38.0)	12,045 (60.3)	<.001
No	1,617(62.0)	7,946 (39.7)	

a Values are expressed as number (%) unless otherwise indicated.

b Determined by using the Rural-Urban Continuum codes (www.ers.usda.gov/ briefing/rurality/ruralurbcon/).

**Table 2.** Logistic Multilevel Model Predicting the Probability of Workplace Secondhand Smoke Exposure in Missouri Based on Individual and County-Level Characteristics^a^

**Fixed Effects**	Odds Ratio (95% Confidence Interval)^b^
Intercept	.10 (.06-.17)
**Individual characteristics**	
**Age**	0.99 (0.984-0.994)
**Male sex**	2.65 (2.23-3.17)
**Race/ethnicity**	
Non-Hispanic white	1 [Reference]
Non-Hispanic black	1.08 (0.83-1.40)
Hispanic	1.03 (0.42-2.48)
Other race	0.77 (0.40-1.48)
**Income^c^**	0.88 (0.82-0.94)
**Education**	
College graduate	1 [Reference]
Some college	2.00 (1.32-3.03)
High school graduate or General Educational Development certificate	2.06 (1.35-3.15)
<High school graduate	2.89 (1.64-5.10)
**Current smoker**	1.91 (1.64-2.23)
**County characteristics^d^**	
Urban area	1 [Reference]
Large rural area	1.16 (0.82-1.66)
Small rural area	1.28 (1.02-1.61)
Isolated	1.66 (1.31-2.11)
**Random effects**	
For intercept, standard deviation	.21
**Model fit**	
Likelihood ratio	χ^2^(3) = 18.0; *P* = .001
Deviance	42,176
Akaike information criterion	42,206

a See the Methods section for details of this analysis.

b Values are expressed as odds ratio (95% confidence interval) unless otherwise indicated.

c Income was treated as continuous.

d Rurality was determined by using the Rural-Urban Continuum codes (www.ers.usda.gov/briefing/rurality/ruralurbcon/).
